# Photocrystallography reveals new metastable nitrosyl linkage isomers in the solid state

**DOI:** 10.1107/S2052252514026980

**Published:** 2015-01-01

**Authors:** Paul R. Raithby

**Affiliations:** a Department of Chemistry, University of Bath, Bath, BA2 7BA, UK

**Keywords:** photocrystallography, light-induced isomerism, reversible isomerism, dinitrosyl compounds

## Abstract

Using the combined techniques of photocrystallography and infrared spectroscopy is a very powerful method for generating and establishing the structures of metastable linkage isomers in the solid state as exemplified by the work of Casaretto *et al.* [*IUCrJ* (2015), **2**, 35–44] on [RuCl(NO)_2_(PPh_3_)_2_]BF_4_.

‘*Photocrystallography*’ is a rapidly developing technique that involves the determination of the full three-dimensional structure of a molecule or array material, using single-crystal X-ray diffraction techniques, while the molecular components are in a photoactivated metastable or short-lived high-energy state (Coppens *et al.*, 2005 ▶). The photoactivation is usually achieved by irradiating the single crystal with a laser or a set of LED lights in the ultra-violet or visible wavelength range. The term ‘*photo­crystallography*’ was originally coined by Philip Coppens, one of the pioneers in the field (Coppens *et al.*, 1998 ▶).

To date, photocrystallography has been used to study the structures of metastable linkage isomers of transition metal coordination complexes, and to investigate the changes in coordination geometry in mononuclear complexes or the length of metal–metal interactions in dinuclear metal complexes (Hatcher & Raithby, 2013 ▶). In a complex that undergoes linkage isomerism, the compound contains one or more ligands that are capable of bonding to the metal centre in more than one way. For example, as elegantly illustrated by Casaretto *et al.* (Casaretto *et al.*, 2015 ▶) the nitrosyl ligand, NO, can coordinate to the metal through the nitrogen atom, through the oxygen atom or in an η^2^-NO sideways bonding mode, and the mode of coordination may switch under photo-irradiation in either the solid state or in solution.

The timescale for the isomerization process is also of importance since this may alter with changes in the crystal environment. At low temperatures the excited photoactivated state may be metastable, lasting longer than the duration of the X-ray experiment, while at higher temperatures the timescale may be reduced to microseconds or even pico­seconds. Using monochromatic synchrotron X-ray radiation and pulsed lasers it has been possible to study the structures of excited state species with microsecond lifetimes using photocrystallographic techniques (Hatcher & Raithby, 2014 ▶). At present, investigations at shorter lifetimes remain the domain of fast spectroscopy. Thus a combination of photocrystallography and time-resolved spectroscopy allows the evolution of structure with time, right across the timescales from picoseconds to the metastable, to be evaluated.

If metastable linkage isomers are to find applications as electronic or data storage materials or as molecular switches or sensors, control over the conversion between the isomers is essential, both in terms of reversibility and the level of conversion achieved under specific photoactivation conditions (Cole, 2011 ▶). Until the late 2000s there were no examples of high levels of conversion between the ground and metastable states, and the factors that would favour the conversion or the stability of the metastable state were not fully understood (Schaniel *et al.*, 2007 ▶). The understanding of the factors that are important has been pioneered by Schaniel and Woike in a series of seminal papers of which Casaretto *et al.* (2015 ▶) is the latest.

Since the photocrystallographic studies of metastable linkage isomers involve single-crystal to single-crystal transformations the unit-cell parameters do not change by more than 2%, as a maximum, otherwise the crystal strain induced would result in crystal deterioration. Thus a key factor in producing crystalline materials capable of undergoing solid-state linkage isomerism under photoactivation is the reduction of crystal stain. This can be achieved by engineering void space into the crystal structures through the use of bulky ligands. Thus, as has been illustrated by Casaretto *et al.* (2015 ▶), for [RuCl(NO)_2_(PPh_3_)_2_]BF_4_ only the bent NO group changes position by re-orientating in the grove formed by the phenyl rings of the two triphenylphosphine groups. Here, the bulk of the phosphines provides a cavity or ‘reaction pocket’ that affords sufficient space for the isomerization to occur without inducing unacceptable crystal strain.

A second condition for the generation of linkage isomers is the presence of a minimum in the excited state potential between the ground state and metastable state. This is dependent on the metal-to-ligand charge transfer processes, and these aspects have been reviewed by Schaniel & Woike (Schaniel & Woike, 2009 ▶). The situation in [RuCl(NO)_2_(PPh_3_)_2_]BF_4_ is similar.

In the reported study of [RuCl(NO)_2_(PPh_3_)_2_]BF_4_ the photcrystallographic investigation is augmented by a detailed infrared study (Casaretto *et al.*, 2015 ▶) which confirms the presence of the major metastable linkage isomer as one with a bent N-bound nitrosyl. This infrared study provides clear evidence for the coordination mode overcoming any ambiguity from the crystallographic study where it is difficult to differentiate between nitrogen and oxygen atoms. In addition, the infrared study allows one of the first kinetic decay studies on a metastable nitrosyl linkage isomer, in the solid state, to be made and shows, that in this example, the kinetics are first order which is similar to that in related ruthenium sulfur dioxide complexes (Sylvester *et al.*, 2014 ▶).

The paper by Casaretto *et al.* (2015 ▶), provides new insights into the photoactivated linkage isomerization process and sets the benchmark for further studies that will lead to the development of electronic devices based on these materials.
